# Transcriptomics of ivermectin response in *Caenorhabditis elegans*: Integrating abamectin quantitative trait loci and comparison to the Ivermectin-exposed DA1316 strain

**DOI:** 10.1371/journal.pone.0285262

**Published:** 2023-05-04

**Authors:** Faruk Dube, Andrea Hinas, Nicolas Delhomme, Magnus Åbrink, Staffan Svärd, Eva Tydén

**Affiliations:** 1 Department of Biomedical Sciences and Veterinary Public Health, Division of Parasitology, Swedish University of Agricultural Sciences, Uppsala, Sweden; 2 Department of Cell and Molecular Biology, Uppsala University, Uppsala, Sweden; 3 Umeå Plant Science Centre (UPSC), Department of Forest Genetics and Plant Physiology, Swedish University of Agricultural Sciences, Umeå, Sweden; 4 Department of Biomedical Sciences and Veterinary Public Health, Section of Immunology, Swedish University of Agricultural Sciences, Uppsala, Sweden; UMass Chan: University of Massachusetts Medical School, UNITED STATES

## Abstract

Parasitic nematodes pose a significant threat to human and animal health, as well as cause economic losses in the agricultural sector. The use of anthelmintic drugs, such as Ivermectin (IVM), to control these parasites has led to widespread drug resistance. Identifying genetic markers of resistance in parasitic nematodes can be challenging, but the free-living nematode *Caenorhabditis elegans* provides a suitable model. In this study, we aimed to analyze the transcriptomes of adult *C*. *elegans* worms of the N2 strain exposed to the anthelmintic drug Ivermectin (IVM), and compare them to those of the resistant strain DA1316 and the recently identified Abamectin Quantitative Trait Loci (QTL) on chromosome V. We exposed pools of 300 adult N2 worms to IVM (10^−7^ and 10^−8^ M) for 4 hours at 20°C, extracted total RNA and sequenced it on the Illumina NovaSeq6000 platform. Differentially expressed genes (DEGs) were determined using an in-house pipeline. The DEGs were compared to genes from a previous microarray study on IVM-resistant *C*. *elegans* and Abamectin-QTL. Our results revealed 615 DEGs (183 up-regulated and 432 down-regulated genes) from diverse gene families in the N2 *C*. *elegans* strain. Of these DEGs, 31 overlapped with genes from IVM-exposed adult worms of the DA1316 strain. We identified 19 genes, including the folate transporter (*folt-2*) and the transmembrane transporter (*T22F3*.*11*), which exhibited an opposite expression in N2 and the DA1316 strain and were deemed potential candidates. Additionally, we compiled a list of potential candidates for further research including T-type calcium channel (*cca-1*), potassium chloride cotransporter (*kcc-2*), as well as other genes such as glutamate-gated channel (*glc-1*) that mapped to the Abamectin-QTL.

## 1 Introduction

Parasitic nematodes cause chronic and debilitating illnesses in humans and animals, as well as economic losses in livestock and agriculture [1–3]. For example, soil-transmitted helminths, infect more than one billion people and cost roughly five million disability-adjusted life years reported in 2010 [4] and plant-parasitic nematodes affecting crops cause annual economic losses of >$80 billion globally [5]. Control strategies for parasitic nematodes in domesticated or livestock animals rely on anthelmintic drugs, which cost the European ruminant livestock industry an estimated €320 million per year [6]. The three major classes of anthelmintic drugs are benzimidazoles, tetrahydropyrimidines, and macrocyclic lactones (MLs). The MLs, such as Ivermectin (IVM) and Abamectin, are the most commonly used drug class in veterinary/human medicine and agriculture due to their high efficacy, low toxicity, and broad-spectrum nature [7]. Glutamine-gated chloride ion channels (GluCls) are the primary target of MLs in nematodes. This consequently leads to the depolarization of pharyngeal muscles and hyperpolarization of body wall muscles [8]. As a result, feeding becomes impaired, and the worm experiences flaccid muscle paralysis [9, 10]. In addition, γ-aminobutyric acid (GABA) receptors have been reported as secondary targets of IVM at concentrations [11] considered therapeutically insignificant.

However, due to the widespread use of anthelmintic drugs, extensive anthelmintic drug resistance has developed in many parasitic nematode species of livestock [12]. Anthelmintic resistance has been reported in *Haemonchus contortus*, *Cooperia* spp., and *Ostertagia ostertagi* infecting sheep in Europe [13]. In addition, anthelmintic-resistant parasitic nematodes in sheep, cattle and goats have been reported in a number of countries, including Brazil, Argentina, the United States, Australia, New Zealand, and South Africa (reviewed [12]). Furthermore, there are growing concerns that mass drug administration (MDA) programs are selecting for anthelmintic resistance among helminths that infect humans [14, 15]. In recent years, there have been emerging reports of IVM resistance in human parasite *Onchocerca volvulus* in Ghana [16] and Cameroon [17]. Evidently, understanding and identifying the genetic markers underlying resistance in parasitic nematodes is necessary.

However, identification of candidate genes or gene variants that drive anthelmintic resistance in parasitic nematodes can be challenging. This is partly due to the fact that parasitic nematodes have a host-dependent life cycle, are difficult to cultivate, maintain, manipulate, and study at scale, and often lack well-annotated genomes in full chromosomes. Until now, only a few parasitic nematodes, have relatively complete chromosomal-level genomes [18–21], allowing genetic and comparative genome analyses. In comparison, the free-living nematode *Caenorhabditis elegans* is biologically simple, has a rapid 3.5-day life cycle, genetically amenable, and molecular tools for manipulation are readily available [22]. In addition, the evolutionary relationship between *C*. *elegans* with parasitic nematodes [23], has led to the adoption of this free-living nematode as a model for parasitic nematodes [22, 24] because it allows for comparative studies and translatable results [25].

Studies in *C*. *elegans* have begun to uncover IVM resistance mechanisms. For example, simultaneous mutations in *glc-1*, *avr-14*, and *avr-15* result in a ~4000-fold resistance to IVM in *C*. *elegans* [26]. These genes, together with *glc-2*, *glc-3*, and *glc-4*, code for the GluCls subunits, which are capable of forming diverse homomeric and heteromeric chloride channels [9, 10]. However, the discovery of IVM-resistant populations of the small-ruminant parasite *H*. *contortus* lacking mutations in the *glc-1*, *avr-14*, and *avr-15* genes [19, 27] suggests the existence of alternative mechanisms of resistance to MLs. Several resistance mechanisms to MLs have been proposed, including changes in gene expression in GluCl and transport protein genes [28]. For example, as previously postulated, decreased expression of drug target genes may result in fewer drug receptors and thus reduced drug efficacy [29, 30]. Gene expression changes of transporter genes of the ATP-binding cassette family, particularly P-glycoproteins (Pgps), also known as efflux pumps, have been linked to anthelmintic drug resistance [31, 32]. This is because efflux pumps eliminate drugs from the cell, preventing them from reaching their target sites. Furthermore, increased expression of genes encoding drug metabolic enzymes, postulated to increase conversion of drugs into inactive metabolites, has been associated with resistance [33]. Collectively, this highlights the multigenic nature and multiple mechanisms of anthelmintic resistance. Although a previous microarray-based study [34] examined the effect of IVM on gene expression in *C*. *elegans*, the strain used was DA1316, which is highly resistant to IVM due to simultaneous mutations in three genes (*glc-1*, *avr-14*, and *avr-15*). As a result, the current study is premised on the lack of a previous transcriptomic study assessing the effect of IVM on gene expression in wild-type (N2) *C*. *elegans*.

In this study, we investigated gene expression in an adult *C*. *elegans* N2 strain exposed to IVM using an RNAseq transcriptomic approach in order to identify the underlying processes and genes involved in the response to IVM in a wild type strain. In addition, we compared our transcriptomic data with microarray data from the IVM-exposed *C*. *elegans* DA1316 strain [34] in order to identify genes that may be uniquely associated with IVM response in IVM-resistant strains. Additionally, we investigated the recently discovered Abamectin Quantitative Trait Loci (QTL) on chromosome V in *C*. *elegans* [35] to determine any potential associations between the identified genes and the QTL.

## 2 Materials and methods

### 2.1 Caenorhabditis elegans exposure and RNA extraction

Adult *C*. *elegans* N2 Bristol strains obtained from the Caenorhabditis Genetics Center were exposed *in vitro* to Ivermectin (IVM). We utilized IVM concentrations of 10^−7^ M and 10^−8^ M, which are within the ranges (10^−9^ M- 10^−6^ M) employed in previous studies [34, 36–40] and the 4 h exposure time was adapted from Laing et al. [34].

A detailed description of the experimental setup is described in [41]. Briefly, synchronized worms at maximum reproduction stage (76 h post L1) were incubated at 20° C in S-complete media supplemented with IVM in concentrations of 10^−7^ and 10^−8^ M (+ 0.025% DMSO final concentration) and 0.025% DMSO (control) for 4 h. The experiment was performed in quadruplicates (~300 worms /replicate). After exposure worms were pelleted by centrifugation, frozen in liquid nitrogen, ground with a pestle and subsequently suspended in Trizol (Invitrogen, Carlsbad, USA). Chloroform was added and the aqueous phase was advanced to NucleoSpin^®^ RNA Plus Kit (Macherey Nagel, Düren, Germany) for RNA extraction. RNA quality and quantity checks were performed using the RNA ScreenTape kit on TapeStation 4150 (Agilent, Santa Clara, USA).

### 2.2 Library preparation and RNA sequencing

The SNP -& SEQ Platform, SciLifeLab Uppsala, Sweden, did the library preparation and sequencing. Using the TruSeq stranded mRNA library preparation kit with polyA selection (Illumina Inc, San Diego, USA); one microgram of RNA from each sample (biological replicate) was used to prepare sequencing libraries. From the libraries, clusters were made, and 150 cycles of paired-end sequencing were done in a single-end SP flowcell using NovaSeq 6000 equipment and v1.5 sequencing chemicals (Illumina Inc., San Diego, USA).

### 2.3 Read processing, mapping and quantification

Ribosomal RNA reads were assessed and filtered out using SortMeRNA (v4.3.6) [42] and rRNA databases from SILVA SSU, LSU (v111) [43] and the RFAM 5/5.8S (v11.0) [44]. Quality trimming and adapter removal was performed using Trimmomatic (v0.39) [45] with the following non-default parameters: sliding window of length four; minimum quality 20; and minimum length of 36. FastQC (v0.11.9) [46] was utilized to evaluate the quality of the resulting reads.

Expression quantification against the *C*. *elegans* transcriptome was performed with Salmon (v1.9.0) [47] using the entire genome (PRJNA13758, release WS283) as the decoy sequence. The transcriptome used was a concatenation of mRNA, ncRNA, and pseudogene files of PRJNA13758, release WS283 available at WormBase [48]. We have made the RNA sequencing raw files available at the ENA database, accessible through the link https://www.ebi.ac.uk/ena/browser/view/ (accession number: PRJEB59331). Additionally, we have uploaded the pipeline for analysis and the necessary files for read processing, mapping, and quantification to the following GitHub repository: https://github.com/ruqse/N2IVM.

R version 4.1.2 was utilized for all R-based analyses. Salmon output was matrix-summarized with tximport R package (v1.22.0) [49] utilizing the TxDb.Celegans.UCSC.ce11.ensGene annotation R package (v3.12.0) [50], for downstream transcript/ gene-level analysis. As a quality control measure, exploratory analysis and visualization was performed on count data.

### 2.4 Principal component analysis

Principal component analysis (PCA) was utilized to investigate sample variation. The variance stabilizing transformation function from the DESeq2 R package (v1.34.0) [51] was applied to count data of genes across all samples (n = 12). Since IVM exposure was performed in batches based on concentration, any batch effects were removed by removeBatchEffect function from the limma R package (v3.50.3) [52]. Using R base prcomp function, principal component analysis was performed on the variance-stabilized transformed (VST) read counts. The top two PCs that account for the most variation in the data were visualized in PCA plot for genes with non-zero read counts using the ggplot2 R package(v3.3.5) [53]. Sample clustering was determined by the enclosing ellipses implemented by the geom_mark_ellipse function in the ggforce R package (https://github.com/thomasp85/ggforce/).

### 2.5 Differential gene expression

Differential gene expression was determined on raw count data by DEseq function in the DESeq2 R package using the following model design, *~batch + IVM concentration*. The batch variable was to compensate for any biases caused by the batch effect. Differentially expressed genes (DEGs) were defined as those with an adjusted *P-*value < 0.05 and Log2foldchange (Log2FC) ≥ 0.5 or ≤ -0.5 (1.4-fold change). These cut-offs are based on recommendations by Schurch et al. [54]. Differentially expressed genes with average expression level (*baseMean*) of <20 across all samples were discarded based on the independent filtering results from DESeq2.

The metadata of DEGs such as concise description, gene ontology association, interacting gene, tissue expression and RNAi/allele phenotype observed were retrieved from WormBase using the SimpleMine tool taking DEGs gene IDs as input. To assess genes shared by each drug concentration, comparative Venn diagrams were constructed using gene IDs and the venn.diagram function of the VennDiagram R package [55]. The get.venn.partition function was utilized to acquire the gene IDs associated with each Venn diagram partition. The hypergeometric test was used to test the significance of the overlapping genes between concentrations using the R base function, phyper.

### 2.6 Over-representation analysis (ORA)

Over-representation analysis was performed on DEGs using all genes (19565) with a non-zero total read count as a background based on Gene Ontology (GO: Release 2021-12-15), Kyoto Encyclopedia of Genes and Genomes (KEGG: Release 2021-12-27) and TRANSFAC (Release 2021.3 classes: v2) databases. The analysis was carried out using the gost function from the gprofiler2 R package (v0.2.1) [56], with default parameters and *C*. *elegans* as the set organism. The output files were processed, and data visualized using ggplot2. Differentially expressed genes from enriched terms putatively involved in drug metabolism/resistance, apoptosis and transcription of regulation were retrieved, categorized, and characterized.

### 2.7 Comparative analysis gene expression between *C*. *elegans* strains N2 and DA1316 after IVM exposure

A comparative analysis of the obtained DEGs with those from a previous study [34] was performed. In that study, *C*. *elegans* strain (DA1316), a triple mutant of the glutamate-gated chloride channel (GluCI) subunits *avr-14*, *avr-15*, and *glc-1* was exposed to IVM 10^−6^ and 10^−7^ M for 4 h. Differential gene expression was determined by microarray assays. To reduce ambiguity prior to comparison, gene names and IDs of DEGs from the previous study were evaluated to determine if they had been renamed, merged, or divided, using the WormBase Gene Name Sanitizer tool. Only DEGs with a fold change of at least 1.4 were included in the comparison and visualized in upsetR plot using the UpSetR R package (v1.4.0) [57]. The hypergeometric test was used to test the significance of the overlapping genes between DA1316 and N2 strain using the R base function, phyper.

### 2.8 Analysis of IVM-induced gene expression within Abamectin-QTL on chromosome V

A recent study described quantitative trait loci (QTL) regions on chromosome V of *C*. *elegans* after exposure to the macrocyclic drug, Abamectin [35]. Based on genome wide association mapping, two regions were detected, left arm (VL) and right arm (VR) located at nucleotides 1,757,246–4,333,001 and 15,983,112–16,599,066, respectively. Linkage mapping approach identified three regions, VL, VR and central (VC) located at 2,629,324–3,076,312, 15,933,659–16,336,743 and 6,118,360–7,374,129, respectively. In our comparative analysis, VL and VR from the genome wide association mapping and VC from the linkage mapping were used. The BSgenome.Celegans.UCSC.ce11genome object from the BSgenome.Celegans.UCSC.ce11 R package (v1.4.2) [58] was used in this analysis. All genes in the VL, VC and VR regions were extracted using the GRanges function and subset based on our DEGs using subsetByOverlaps function. Both functions are derived from GenomicRanges R package (v1.46.1) [59]. To eliminate any chromosome length biases, the distribution/ enrichment of DEGs across all chromosomes was estimated using the Fisher’s Exact Test for Count Data in R. Differentially expressed genes mapping to the QTL were subsequently identified and visualized on a karyoplot from karyoploteR R package (v1.20.3) [60].

## 3 Results

### 3.1 Transcriptomic variation amongst adult *C*. *elegans* worms exposed to Ivermectin

Total RNA (RIN > 8) was sequenced from *C*. *elegans* adults at maximum reproduction exposed to IVM 10^−7^ and 10^−8^ M yielding sequencing data of 21–60 million read-pairs/sample. The used concentrations of IVM treatment were selected according to [41]. However, due to the very low number of differentially expressed genes in IVM 10^−9^ M, only data from IVM 10^−7^ and 10^−8^ M was used in the current study. Following quality control and evaluation with SortMeRNA and Trimmomatic, 16–42 million read-pairs/sample were obtained with a mean phred score above 33, indicating high quality reads. The read-mapping rate to the custom-built PRJNA13758 *C*. *elegans* transcriptome (see Methods) and the *C*. *elegans* genome (PRJNA13758, release WS283) as a decoy, was 97% using Salmon.

**Fig 1:**
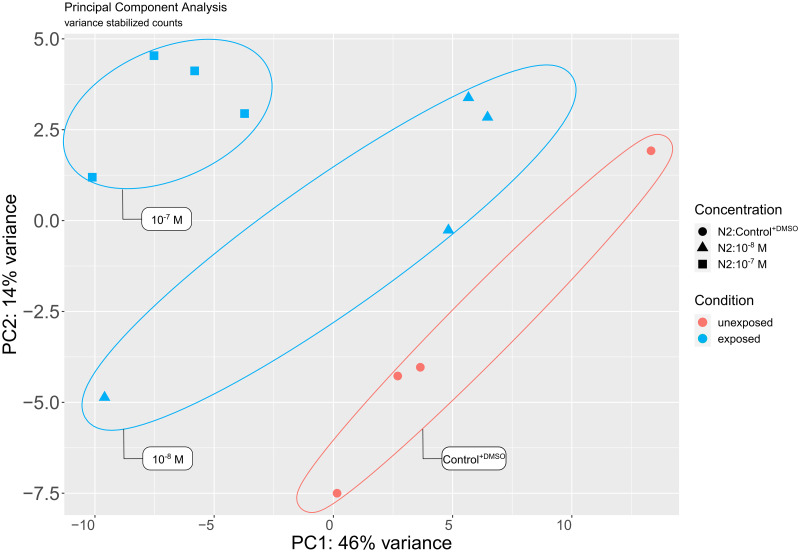
Variation in gene expression among worms exposed to IVM. A PCA plot of *C*. *elegans* N2 strain based on variance stabilized read counts from worm pools (n = 300) after exposure to IVM 10^−7^ M, 10^−8^ M or control ^+DMSO^. Pools are separated by both PC1 and PC2, which represent the largest variances in the data and are ellipsed by concentration/treatment.

According to principal component analysis of the VST read counts, worm pools (n = 300 worms per pool), both PC1 and PC2 explained the separation by treatment condition (Fig 1).

### 3.2 Differential gene expression

**Fig 2:**
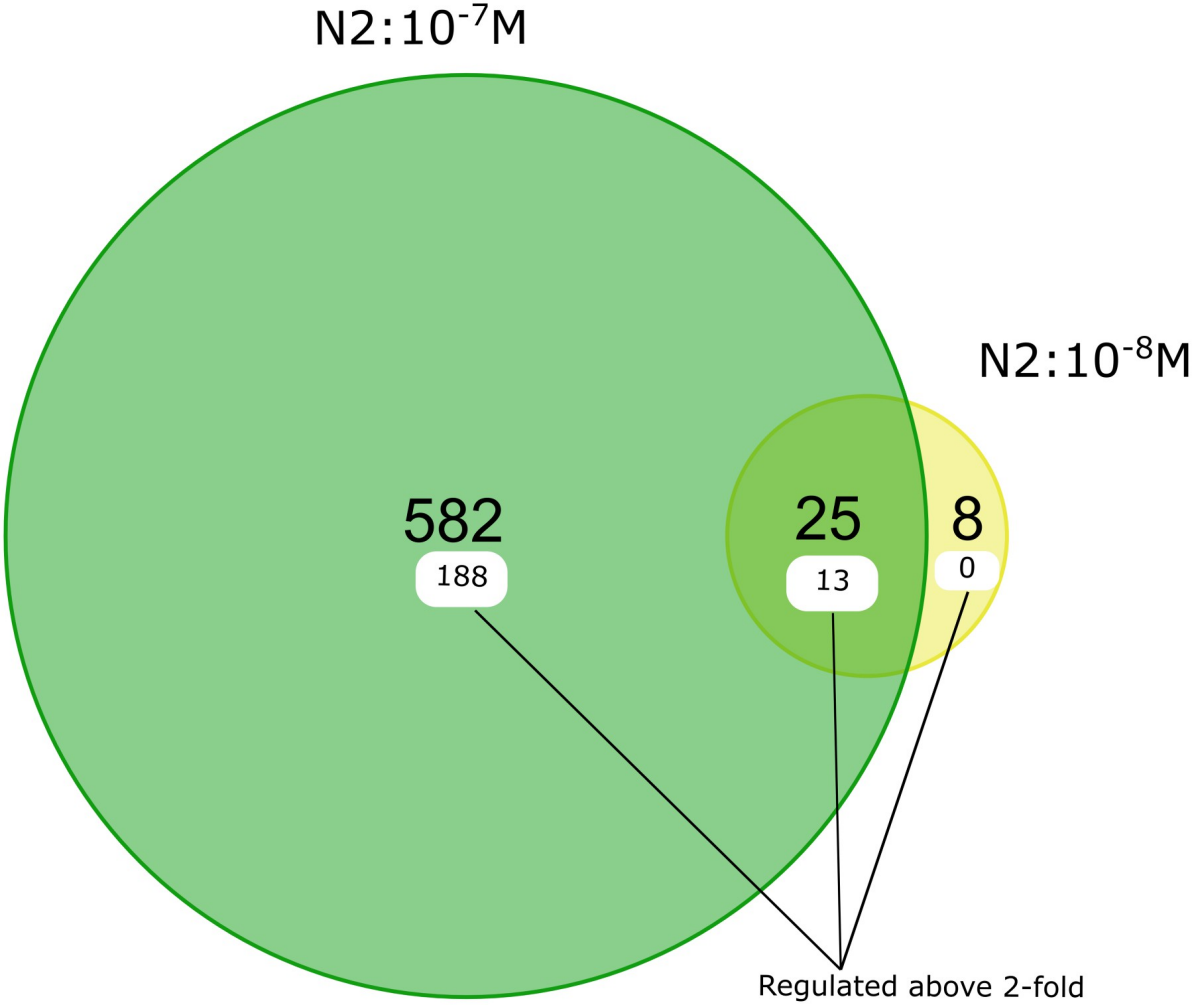
Differentially expressed in IVM-exposed *C*. *elegans* N2 strain. Venn diagrams showing the number of DEGs in *C*. *elegans* N2 strain between IVM concentrations 10^−7^ and 10^−8^ M based recommendations by Schurch et al. [54]. The number of genes differentially expressed above 2-fold are labelled in white.

Following DESeq2 analysis, 615 genes were differentially expressed after IVM 10^−7^ M and/or 10^−8^ M exposure (S1 Table). Of these, 76% (468 genes) had known functional annotations including GO annotations according to WormBase (release WS283). Ninety five percent (582 genes) of DEGs were exclusively detected in 10^−7^ M, 1% (8 genes) in 10^−8^ M and 4% (25 genes) showed significant (*p*-value = 0.00001) overlap between the two concentrations (Fig 2 & S2 Table). About 33% (201 genes) of DEGs were altered more than 2-fold, with 85 genes upregulated and 116 genes downregulated. The top ten up- and downregulated DEGs consisted of a diverse set of genes or gene families including transmembrane transporters, and heat shock proteins (S3 Table).

**Fig 3:**
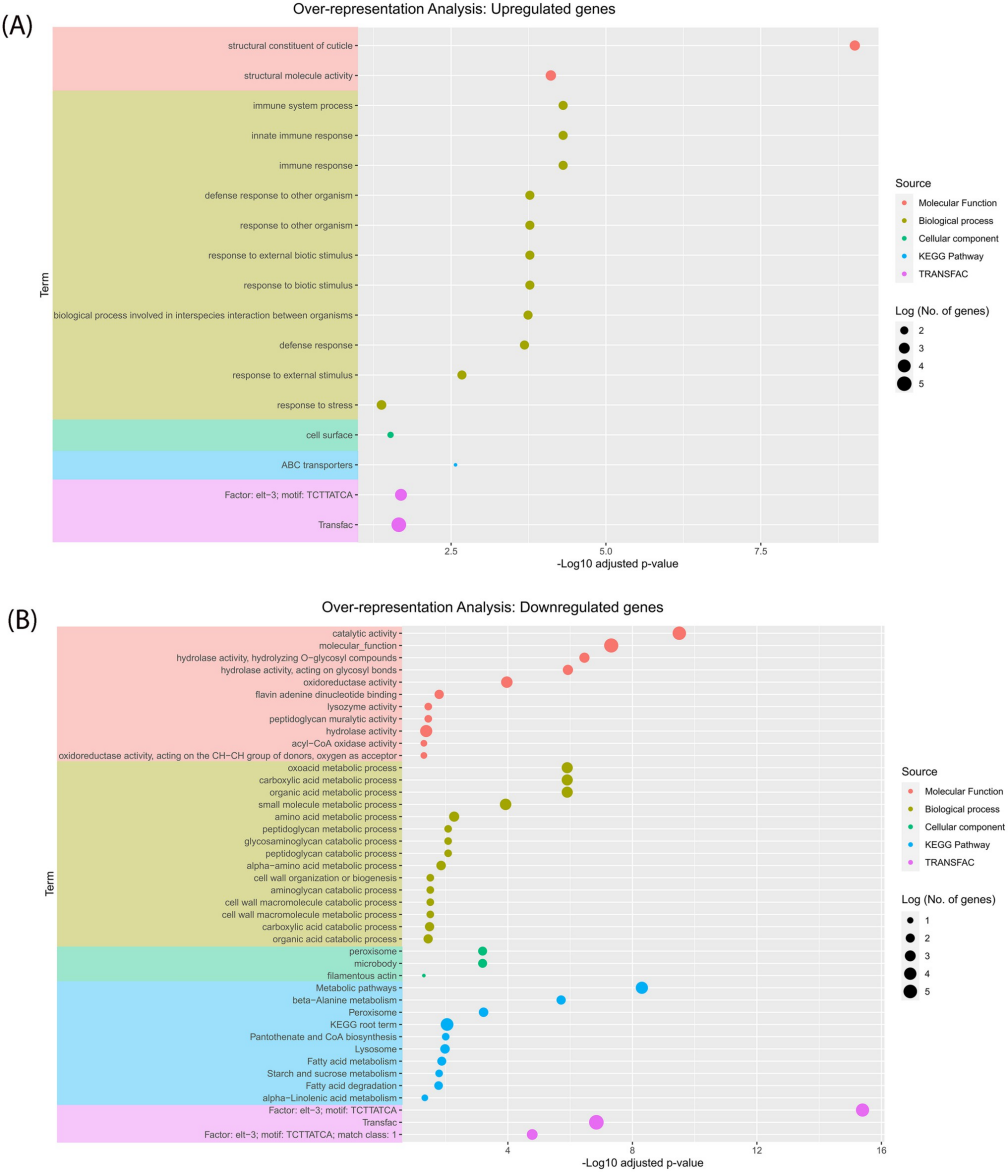
**A.** Biological processes underpinned by upregulated genes. An over-representation plot showing significantly enriched for terms among upregulated DEGs based on Gene ontology (molecular function, biological process and cellular component), Kyoto Encyclopedia of Genes and Genomes (KEGG), and TRANSFAC databases. The dots represent an enriched term. **B.** Biological processes underpinned by downregulated genes. An over-representation plot showing significantly enriched for terms among downregulated DEGs based on Gene ontology (molecular function, biological process and cellular component), Kyoto Encyclopedia of Genes and Genomes (KEGG), and TRANSFAC databases.

To gain insights into the biological pathways underlying the differentially expressed genes, we conducted over-representation analysis on both the upregulated (Fig 3A & S4 Table) and downregulated genes (Fig 3B & S4 Table). We found that the downregulated DEGs exhibited more enrichment terms (42) compared to the upregulated genes (17). The ORA on the upregulated DEGs revealed enrichment of terms related to response to external stimulus, ABC-transport activity, and Factor: elt-3; motif: TCTTATCA (TF: M07154) based on GO, KEGG pathway, and TRANSFAC databases, respectively (Fig 3A & S4 Table). In contrast, ORA on the downregulated genes showed enrichment of terms related to metabolism of glycans, amino acids, and organic compounds, Factor: elt-3; motif: TCTTATCA (TF: M07154) among others, based on the same databases (Fig 3B & S4 Table). Differentially expressed genes from ORA terms with putative involvement in drug metabolism were explored further.

#### 3.2.1 Differentially expressed genes encoding drug receptors

After exposure of N2 worms to IVM 10^−7^ M, the putative drug target *glc-1*, an alpha subunit of the GluCI receptor, *glc-1* was downregulated 1.4-fold. In addition, the putative drug target, *lgc-26*, a possible nicotinic acetylcholine receptor was downregulated 1.6-fold after exposure to IVM 10^−7^ M (Table 1). However, in IVM 10^−8^ M concentration, differential expression of the putative drug receptors was not observed.

**Table 1:** Upregulated genes putatively involved in IVM response in *C*. *elegans* N2 strain.

Category	Gene	LFC:N2:10^−7^ M^a^	-Log10 Adjusted p-value	LFC:N2:10^−8^ M^b^	-Log10 Adjusted p-value	Description^c^
***Phase I metabolism***	*cyp-13B2*	0.56	1.88			cytochrome P450
	*cyp-33C8*	1.29	7.43			cytochrome P450
	*cyp-34A8*	0.73	2.11			cytochrome P450
***Phase II metabolism***	*ugt-62*	0.81	1.55			UDP-glycosyltransferase
***Ion channels***	*atp-6*	1.88	34.40	2.06	46.06	ATP synthase subunit a
	*best-21*	0.69	1.75			chloride channel activity
	*F58G6*.*9*	1.62	1.62			copper ion transmembrane transporter activity
	*kvs-5*	1.10	2.84			potassium ion transmembrane transport
***Solute transporters***	*pgp-5*	1.83	1.38			P-glycoprotein ATP-dependent efflux pump
	*pgp-6*	1.15	1.93			P-glycoprotein ATP-dependent efflux pump
	*pgp-9*	1.52	4.61			P-glycoprotein ATP-dependent efflux pump
	*folt-2*	1.92	1.94			folate transporter
	*pmp-4*	1.05	2.11			long-chain fatty acid transporter
***Transcription factors***	*fkh-3*	0.52	1.71			forkhead transcription factor
	*fkh-4*	0.58	1.50			forkhead transcription factor
	*mdl-1*	0.50	2.99			basic helix-loop-helix (bHLH) protein
	*nhr-17*	0.55	1.75			nuclear receptor super family
	*nhr-57*	0.58	1.54			nuclear receptor super family
	*nhr-115*	1.69	1.42			nuclear receptor super family
	*nhr-137*	0.53	1.30			nuclear receptor super family
	*tbx-33*	0.58	1.83			T-box transcription factor
	*tbx-38*	0.99	1.33			T-box transcription factor
	zip-3	0.86	2.53			bZip transcription factor
***Involved in apoptosis***	*pal-1*	0.65	8.25	0.54	5.23	homeodomain protein

Alphabetically sorted upregulated genes putatively characterized as drug targets or involved in metabolite transport, xenobiotic metabolism, gene expression regulation, and apoptosis processes

^a^Log2Foldchange for Ivermectin drug concentration 10^−7^ M

^b^Log2Foldchange for Ivermectin drug concentration 10^−8^ M

^c^Full description can be found in S1 Table

#### 3.2.2 Differentially expressed genes involved in Phase I and II xenobiotic metabolism

There were 11 DEGs putatively classified to be involved in Phase I metabolism, of which eight were downregulated (Table 2) and three were upregulated in IVM 10^−7^ M concentration (Table 1). Eight genes were attributed to the cytochrome P450 family, of which three genes, *cyp-33C8*, *cyp-34A8* and *cyp-13B2* were on average upregulated 1.8-fold while the rest, *cyp-33E1*, *cyp-13A5*, *cyp-25A1*, *cyp-35A2* and *cyp-34A9* were downregulated between 1.5 and 2.2-fold. Other genes involved in drug metabolism with differential expression included Flavin-containing monoxygenase, *fmo-2*, short chain dehydrogenase, *dhs-7* and aldehyde dehydrogenase *adh-5*, which were downregulated between 1.5 and 6-fold.

**Table 2:** Downregulated genes putatively involved in IVM response in *C*. *elegans* N2 strain.

Category	Gene	LFC:N2:10^−7^ M^a^	-Log10 Adj. p-value	LFC:N2:10^−8^ M^b^	-Log10 Adj. p-value	Description^c^
***Drug target***	*glc-1*	-0.50	2.18			alpha subunit of a glutamate-gated chloride channel
	*lgc-26*	-0.71	1.55			nicotinic acetylcholine receptor-like LGIC
***Phase I metabolism***	*alh-5*	-0.90	2.17			aldehyde dehydrogenase (NAD+) activity
	*cyp-13A5*	-0.74	1.85			cytochrome P450
	*cyp-25A1*	-0.87	1.43			cytochrome P450
	*cyp-33E1*	-0.54	2.35			cytochrome P450
	*cyp-34A9*	-1.12	6.73			cytochrome P450
	*cyp-35A2*	-0.99	9.22			cytochrome P450
	*dhs-7*	-2.60	6.50			regulation of reactive oxygen species metabolic process
	*fmo-2*	-1.25	6.57			flavin-containing monoxygenase
***Phase II metabolism***	*gst-20*	-1.02	3.67			glutathione transferase
	*gst-4*	-0.56	1.33			glutathione transferase
	*gst-42*	-0.60	4.21			glutathione transferase
	*gst-6*	-0.86	1.62			glutathione transferase
	*gstk-2*	-0.63	1.68			kappa class glutathione transferase
	*ugt-11*	-0.71	1.62			UDP-glycosyltransferase
	*ugt-12*	-0.64	1.34			UDP-glycosyltransferase
	*ugt-22*	-0.91	3.56			UDP-glycosyltransferase
	*ugt-43*	-1.61	4.48			UDP-glycosyltransferase
	*ugt-44*	-1.20	8.77			UDP-glycosyltransferase
***Ion channels***	*atp-6*	1.88	34.40	2.06	46.06	ATP synthase subunit a
	*best-1*	-1.29	4.21			chloride channel activity
	*best-5*	-1.06	2.37			chloride channel activity
	*catp-1*	-0.51	2.06			alpha subunit of the Na+/K+- and H+/K+-pump P-type ATPase family
	*cca-1*	-0.53	1.54			calcium channel alpha subunit
	*F47E1*.*4*	-0.89	1.97			sodium-independent organic anion transmembrane transporter activity
	*kcc-2*	-0.53	3.46			potassium chloride cotransporter
	*ncx-2*	-0.62	1.43			3Na[+]/1Ca[2+] exchanger
	*nlr-1*	-0.63	1.71			Neurexin Like receptor
	*tmc-2*	-0.92	1.42			mechanosensitive ion channel activity
	*Y70G10A*.*3*	-0.55	5.24			sodium-independent organic anion transmembrane transporter activity
	*ZK185*.*5*	-0.60	2.71			cation transmembrane transporter activity
***Solute transporters***	*aat-3*	-0.50	3.33			amino acid transporter catalytic subunit
	*amt-4*	-0.91	3.73			ammonium transporter protein family
	*aqp-1*	-1.07	3.43			aquaglyceroporin
	*C13C4*.*6*	-1.20	4.82			transmembrane transporter activity
	*F11A5*.*9*	-0.52	1.62			transmembrane transporter activity
	*F17C11*.*12*	-0.52	3.28			transmembrane transporter activity
	*F23F12*.*13*	-1.63	2.10			transmembrane transporter activity
	*gem-1*	-0.65	2.26			monocarboxylic acid transmembrane transporter activity
	*glt-5*	-0.93	4.85			glutamate/aspartate and neutral amino acid transporter
	*haf-9*	-0.65	2.04			half-type ATP-binding cassette (ABC) transporter
	*hmit-1*.*3*	-0.73	2.36			(H+)-dependent myo-inositol transporter
	*K09C4*.*1*	-0.92	1.33			hexose transmembrane transporter activity
	*M162*.*5*	-0.77	2.72			transmembrane transporter activity
	*pept-1*	-0.87	2.05			low affinity/high capacity oligopeptide transporter
	*pmp-1*	-0.77	1.57			ATP-binding cassette (ABC) transporter
	*slc-25A29*	-0.78	2.57			high-affinity L-arginine transmembrane transporter activity
	*slc-28*.*1*	-1.19	4.21			nucleoside:sodium symporter activity
	*slc-36*.*5*	-0.62	2.31			amino acid transmembrane transporter activity
	*spp-1*	-0.55	10.83			saposin (B) domain-containing caenopore
	*swt-1*	-0.50	13.61			sugar transmembrane transporter activity
	*T07G12*.*5*	-2.89	3.19			L-ascorbic acid transmembrane transporter activity
	*T22F3*.*11*	-2.81	2.22			transmembrane transporter activity
	*wht-7*	-1.03	4.12	-0.74	2.68	ABC-type transporter activity
	*Y4C6B*.*3*	-0.59	2.30			transmembrane transporter activity
	*ZK550*.*2*	-0.96	1.77			transmembrane transporter activity
***Transcription factors***	*bar-1*	-0.75	1.87			beta-catenin transcription coactivator
	*ceh-31*	-1.11	3.11			homeobox domain protein
	*egl-43*	-1.28	2.09			zinc finger protein
	*elt-2*	-0.69	6.04			GATA-type transcription factor
	*fkh-6*	-0.84	1.93			forkhead transcription factor
	*ham-2*	-0.95	1.52			C2H2 zinc finger-containing protein
	*let-381*	-0.95	2.15			forkhead transcription factor
	*lin-22*	-1.09	1.99			basic helix-loop-helix (bHLH)-containing protein
	*M03D4*.*4*	-0.79	6.49			DNA-binding transcription factor activity
	*myrf-2*	-0.84	4.20	-0.78	1.42	glutamine/asparagine-rich domain protein
	*nhr-21*	-0.77	1.52			nuclear receptor super family
	*nhr-55*	-1.46	3.84			nuclear receptor super family
	*nhr-99*	-1.04	2.21			nuclear receptor super family
	*nhr-119*	-0.69	1.80			nuclear receptor super family
	*nhr-173*	-0.89	1.51			nuclear receptor super family
	*odd-2*	-0.66	3.78			ODD-SKIPPED family
	*pros-1*	-0.97	2.24			homeodomain protein
	*sta-1*	-0.70	3.17			STAT family transcription factor
	*tbx-2*	-0.51	4.91			T-box transcription factor
	*ttx-1*	-0.90	1.84			OTD/OTX hoemeodomain protein
	*unc-62*	-0.80	1.54			Meis-class homeodomain protein
	*unc-98*	-0.76	2.56			C2H2 zinc finger protein
	*unc-120*	-0.62	3.87			MADS-box transcription factor
	*unc-130*	-0.64	1.82			forkhead domain transcription factor
***Involved in apoptosis***	*ces-1*	-1.22	8.81			C2H2-type zinc finger transcription factor
	*ces-2*	-0.67	2.65			basic region leucine-zipper (bZIP) transcription factor
	*lec-6*	-0.56	2.18			’proto’ type galectin (beta-galactosyl-binding lectin)
	*T05C3*.*6*	-1.00	1.55			paralog of T05C3.2

Alphabetically sorted downregulated genes putatively characterized as drug targets or involved in metabolite transport, xenobiotic metabolism, gene expression regulation, and apoptosis processes

^a^Log2Foldchange for Ivermectin drug concentration 10^−7^ M

^b^Log2Foldchange for Ivermectin drug concentration 10^−8^ M

^c^Full description can be found in S1 Table

Twelve DEGs putatively identified as UDP-glycosyltransferases and glutathione S-transferases involved in Phase II metabolism were enriched exclusively in IVM 10^−7^ M (Tables 1 & 2). UDP-glycosyltransferases, *ugt-62* was upregulated (Table 1) above 1.8-fold while *ugt-11*, *ugt-12*, *ugt-22*, *ugt-43* and *ugt-44* were downregulated between 1.4 and 3-fold (Table 2). In addition, glutathione S-transferases, *gst-4*, *gst-6*, *gst-20*, *gst-42* and *gstk-2* were downregulated between 1.5 and 2-fold. Only one gene, *ugt-22*, appeared in 10^−8^ M, and was downregulated 1.5-fold.

#### 3.2.3 Differentially expressed genes involved in ion transporter activity

There were 15 DEGs putatively identified or confirmed as ion channels (Tables 1 & 2). Genes *best-1*, *best-5*, *catp-1*, *cca-1*, *F47E1*.*4*, *kcc-2*, *ncx-2*, *nlr-1*, *tmc-2*, *Y70G10A*.*3*, and *ZK185*.*5*, were downregulated between 1.4- and 2.4- fold (Table 2). Conversely, genes *atp-6*, *best-21*, *F58G6*.*9* and *kvs-5* were upregulated on average 2-fold (Table 1).

#### 3.2.4 Differentially expressed genes involved in other solute transmembrane transporter activity

There were 30 DEGs involved in transmembrane transport and comprised of various genes and gene families (Tables 1 & 2). The DEGs consisted predominantly of P-glycoprotein efflux pumps (3 genes), other solute transporters (27 genes). Efflux pumps included, *pgp-5*, *pgp-6*, and *pgp-9*, appeared exclusively in IVM 10^−7^ M and, were all upregulated above 2-fold (Table 1). Other solute transporters included folate transporter (*folt-2*), long-chain fatty acid transporter (*pmp-1* and *pmp-4*), amino acid transporter (*aat-3*, *slc-25A29*, *slc-36*.*5*, *pept-1*, *glt-5*, and *slc-28*.*1*), myo-inositol transport (*hmit-1*.*3*), sugar transmembrane transporter (*swt-1*, *K09C4*.*1*) etc. Of these, only *folt-2* and *pmp-4* were upregulated (Table 1).

#### 3.2.5 Differentially expressed genes involved in transcriptional regulation of xenobiotic metabolism

The differential screening identified 35 transcriptional factors (Tables 1 & 2), of these, nine were nuclear hormone receptors (NHRs) and one was a GATA-type transcription factor. These ten have been postulated to be involved in xenobiotic metabolism (see review [61]). The NHRs, *nhr-17*, *nhr-57* and *nhr-115*, *nhr-137* were upregulated between 1.4 and 3.2-fold (Table 1) whereas *nhr-21*, *nhr-55*, *nhr-99*, *nhr-119*, *nhr-121* and *nhr-173* were downregulated between 1.6 and 2.8-fold. The GATA transcription factor, *elt-2* was downregulated 1.6-fold (Table 2).

#### 3.2.6 Differentially expressed genes involved in apoptosis

**Fig 4:**
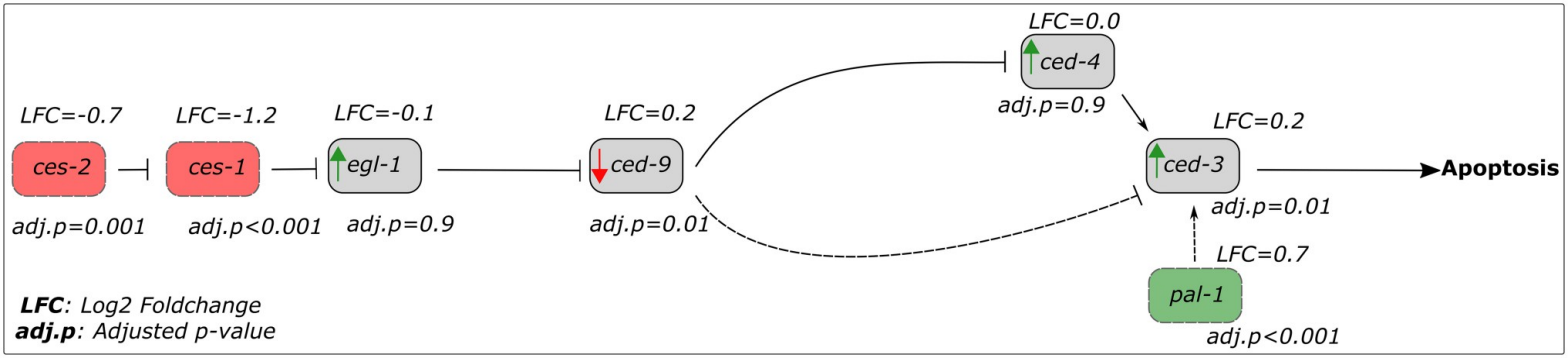
Adapted illustration of the genetic pathway of apoptosis from Conradt and Xue [62]. The boxes symbolize genes, where red and green boxes represent apoptotic genes that in the current study were downregulated or upregulated, respectively according to our thresholds (see Methods). Solid and dashed T-shaped lines represent repression of gene expression, whereas solid and dashed black arrows represent the opposite. Hypothetically, the green and red arrows represent the respective gene’s upregulation and downregulation consequent of T-lines and black arrows.

The Ivermectin concentration of 10^−7^ M was found to exhibit a greater number of differentially expressed genes (*ces-1*, *ces-2*, *lec-6*, *T05C3*.*6*, and *pal-1*) involved in apoptosis compared to the concentration of 10^−8^ M (*pal-1*) (Tables 1 & 2). Four of these DEGs (*ces-1*, *ces-2*, *lec-6*, and *T05C3*.*6*) were observed to be downregulated between 1.4 and 2.3-fold at IVM 10^−7^ M (Table 2). C2H2-type zinc finger transcription factor *ces-1* and basic region leucine-zipper (bZIP) transcription factor *ces-2* have been previously reported as repressors of the gene *egl-1*, a key activator of apoptosis in *C*. *elegans* [62]. The presence of caudal-type homeodomain transcription factor *pal-1*, an activator of the apoptotic gene *ced-3* (Fig 4), was detected in both IVM 10^−7^ M and 10^−8^ M and was found to be equally upregulated (~ 1.5-fold) in both concentrations (Table 1). This suggests that IVM (at 10^−7^ M and 10^−8^ M) partially activates the apoptotic machinery in the N2 *C*. *elegans* strain.

### 3.3 Comparative analysis of gene expression in the *C*. *elegans* strains N2 and DA1316 following exposure to IVM

We compared our transcriptomic data from the *C*. *elegans* strain N2 to microarray data obtained from the IVM-resistant *C*. *elegans* strain DA1316 in a previous study [34]. Based on our preset criteria (see Methods), 152 genes were differentially expressed in the DA1316 strain after IVM exposure for 4 h. Ninety-one percent (139 genes) of DEGs were exclusively detected in 10^−6^ M, 7% (11 genes) in10^-7^ M and 1% (2 genes) occurred in both concentrations (S1 Fig).

**Fig 5:**
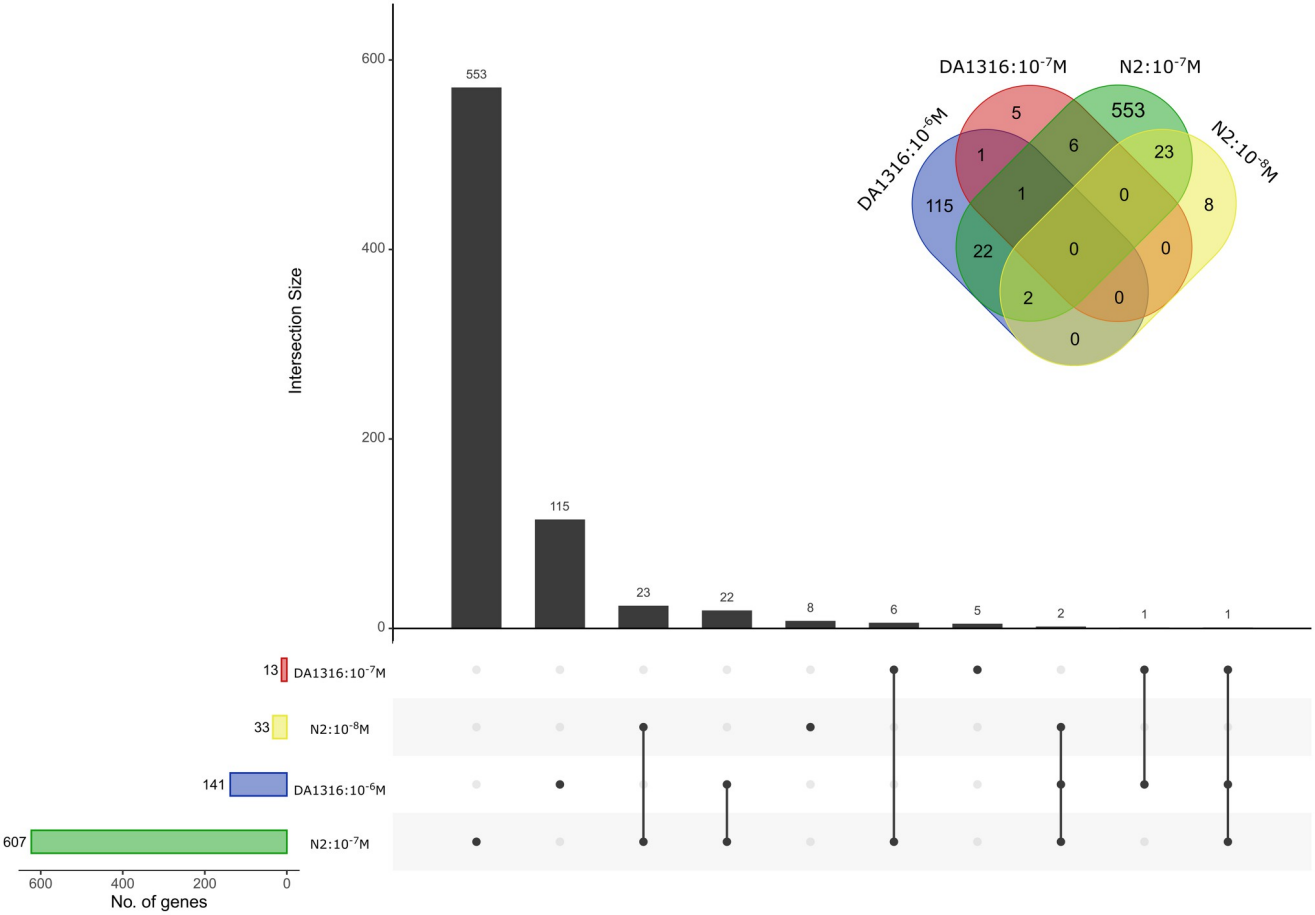
Differentially expressed genes in IVM-exposed *C*. *elegans* N2 and DA1316 strains. An UpSetR plot and Venn diagrams displaying the number of differentially expressed genes that overlap within and between *C*. *elegans* strains N2 and DA1316 [34] exposed to IVM 10^−7^ M, 10^−8^ M and 10^−6^ M, 10^−7^ M, respectively. The black boxes indicate the number of genes contained within each partition.

Overall, irrespective of IVM concentration, comparison of DEGs between DA1316 and N2 strains revealed that the N2 strain showed four times more differentially expressed genes. Thirty-one genes showed significant overlap (*p*-value < 0.00001) between the two strains (Fig 5 & Table 3). Of these, 61% (19 genes) showed an opposite expression i.e., upregulation in one strain and downregulation in another, notably folate transporter gene, *folt-2* and putative transmembrane transporter, *T22F3*.*11* (Table 3 & S5 Table). Gene *folt-2* was downregulated 6-fold and 2-fold in DA1316: 10^−6^ and DA1316: 10^−7^, respectively, and upregulated 4-fold in N2:10^−7^ M concentration (S5 Table). Similarly, *T22F3*.*11* was upregulated 3-fold in DA1316: 10^−7^ M and downregulated 7-fold in N2:10^−7^ M concentration. Eighteen of the 31 genes were enriched for Phase II metabolic, transmembrane transport and stress response processes. Twelve of the shared genes (*acox-1*.*5*, *cpt-5*, *drd-5*, *F42A10*.*7*, *gst-4*, *pud-1*.*2*, *pud-2*.*1*, *pud-3*, *pud-4*, *sams-1*, *ugt-12* and *ugt-22*) were downregulated in both strains. The majority of the shared DEGs (22 genes) occurred in DA1316: 10^−6^ M and N2:10^−7^ M concentrations, whereas six genes (*mtl-1*, *C23G10*.*11*, *scl-2*, *F21C10*.*10*, *T22F3*.*11* and *adh-1*) occurred in DA1316: 10^−7^ M and N2:10^−7^ M concentrations (Fig 5 & S5 Table).

**Table 3:** Overlapping differentially expressed genes between IVM-exposed *C*. *elegans* N2 and DA1316 strain.

*Gene*	LFC:N2:10^−7^ M^a^	LFC:N2:10^−8^ M^b^	LFC:DA1316:10^−6^ M^c^	LFC:DA1316:10^−7^ M^d^	Description
***acox-1***.***5***	-0.89		-0.72		acyl-CoA oxidase
***adh-1***	-1.30			1.07	alcohol dehydrogenase
***C09B8***.***4***	0.59		-1.20		integral component of membrane
***C23G10***.***11***	-3.60			1.23	N.A.
***C35A5***.***3***	1.29		-1.83		transmembrane transporter
***cpt-5***	-1.05		-0.71		acyltransferase
***dod-19***	0.50		-0.99		innate immune response
***drd-5***	-0.63		-0.84		oxidoreductase activity
***F19B2***.***5***	2.18		-1.39		ATP-dependent chromatin remodeler
***F21C10***.***10***	-0.88			1.10	N.A
***F42A10***.***7***	-0.78		-0.73		N.A.
***F58G6***.***9***	1.62		-1.72		copper ion transmembrane transport
***folt-2***	1.92		-2.55	-1.17	folate transporter
***gst-4***	-0.56		-1.41		glutathione transferase
***hsp-17***	2.04		-0.79		heat shock protein
***msra-1***	0.85		-1.08		methionine sulfoxide-S-reductase
***mtl-1***	-0.63			1.59	metallothioneins, small, cysteine-rich, metal-binding protein
***pud-1***.***2***	-0.96		-0.58		N.A.
***pud-2***.***1***	-0.83		-0.59		N.A.
***pud-3***	-2.13		-1.13		N.A.
***pud-4***	-0.73		-0.66		N.A.
***rips-1***	0.87		-1.04		S-adenosyl-L-methionine-dependent methyltransferase
***sams-1***	-0.84		-1.02		S-adenosyl methionine synthetase
***scl-2***	-0.96			1.31	sperm coating protein
***T05E12***.***6***	0.86		-1.23		N.A.
***T13F3***.***6***	1.13		-0.81		N.A.
***T22F3***.***11***	-2.81			1.44	transmembrane transport"
***ugt-12***	-0.64		-0.79		UDP-glycosyltransferase
***ugt-22***	-0.91	-0.61	-0.77		UDP-glycosyltransferase
***Y94H6A***.***10***	1.31	0.64	-1.27		N.A.
***ZK228***.***4***	2.58		-1.01		N-acetyltransferase

Alphabetically sorted differentially genes that overlap between IVM-exposed *C*. *elegans* N2 and DA1316 strain. Genes in bold showed an opposite expression i.e., upregulation in one strain and downregulation in another.

^a^Log2Foldchange for Ivermectin drug concentration 10^−7^ M in the N2 strain

^b^Log2Foldchange for Ivermectin drug concentration 10^−8^ M in the N2 strain

^c^Log2Foldchange for Ivermectin drug concentration 10^−7^ M in the DA1316 strain

^d^Log2Foldchange for Ivermectin drug concentration 10^−8^ M in DA1316 strain

### 3.4 Ivermectin-induced DEGs are mapped within the Abamectin QTL on chromosome V

**Fig 6:**
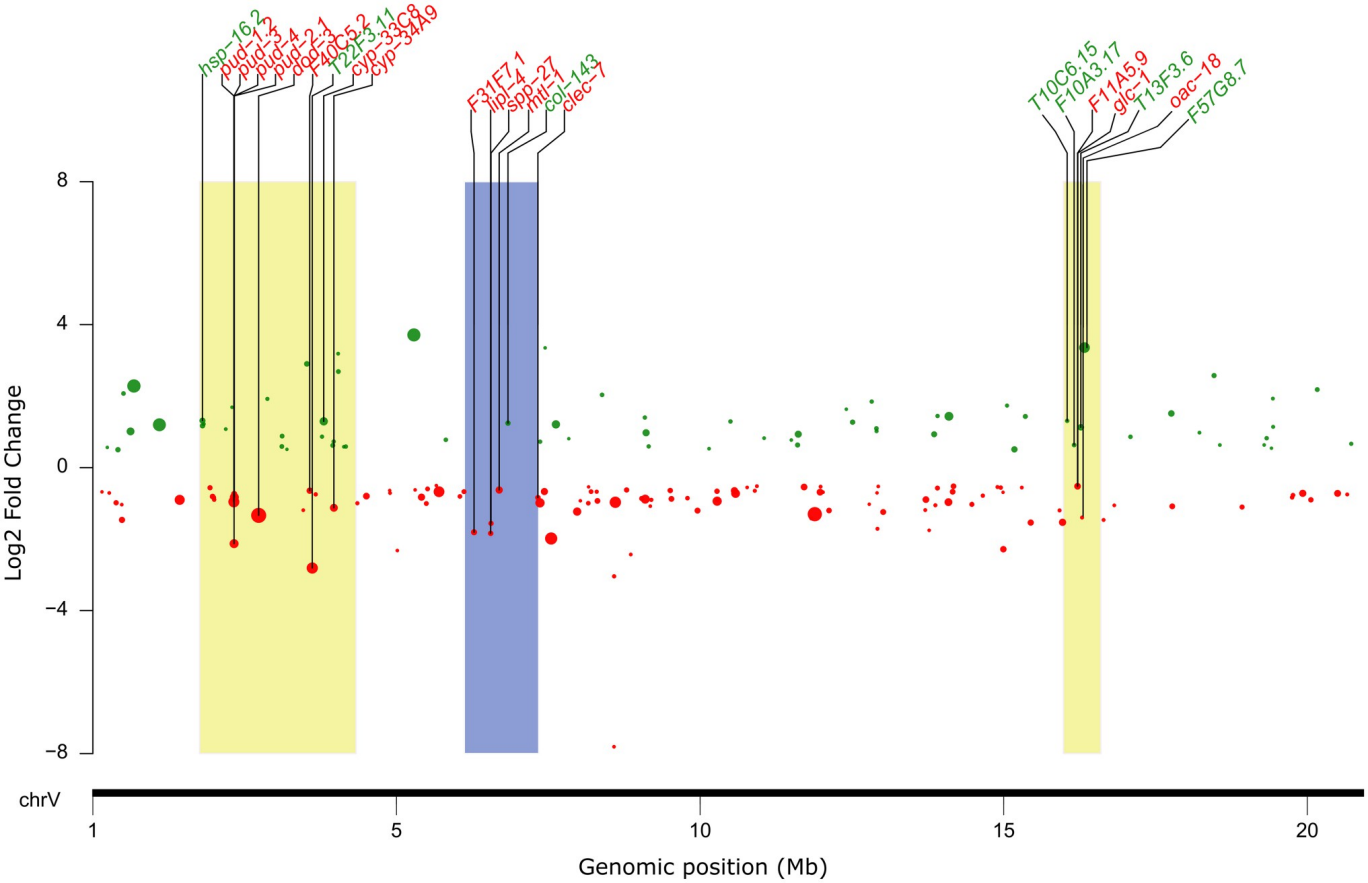
Differentially expressed genes of IVM-exposed *C*. *elegans* N2 strain map to Abamectin-QTL on chromosome V. The DEGs are represented by green (upregulated) and red (downregulated) dots, with the size of the dot representing the -log10 (*adj*. *p-value*) of expression, where the larger the dot, the more significantly expressed. The x-axis represents chromosome V and the y-axis represents the Log2 fold change. The karyoplot shows DEGs (labeled in green and red) mapped to abamectin-QTL VL, VC, and VR represented by yellow, blue, and yellow boxes, respectively.

Evans et al. [35] identified three major QTL (VL, VR and VC) correlated to natural variation in Abamectin response, on chromosome V in *C*. *elegans*. We decided to use this information to see if any of the DEGs induced by IVM, map in the QTL regions. There was significant (adjusted *p =* 0.01) enrichment for DEGs in chromosomes X and V (S6 Table). A comparison of the DEGs of IVM-exposed *C*. *elegans* N2 adult worms to the QTL revealed that 45 DEGs mapped to the three QTL, 51% (23 genes) were upregulated, and the majority (71%) mapped to the VL locus (Fig 6 & S7 Table). Of the 45 DEGs, nine (*folt-2*, *mtl-1*, *pud-1*.*2*, *pud-2*.*1*, *pud-3*, *pud-4*, *rips-1*, *T13F3*.*6* and *T22F3*.*11*) overlap with microarray data from IVM-resistant strain (DA1316) [34]. In addition, eight other DEGs from the DA1316 strain mapped to the QTL (S8 Table). The VL locus contained 32 DEGs (S7 Table) from N2 strain, which included seven metabolic genes (*cyp-33C8*, *cyp-34A9*, *acs-3*, *T06A1*.*5*, *dach-1*, *alh-5*, *cyp-34A8*), six transport-related genes (*dod-3*, *T22F3*.*11*, *Y39H10B*.*2*, *folt-2*, *Y32G9B*.*1*, *slc-28*.*1*), six stress response genes (*hsp-16*.*2*, *irg-1*, *hsp-16*.*41*, *Y73C8C*.*3*, *irg-2*, *Y73C8C*.*8*), two transcription factors (*nhr-57*, *nhr-115*), a muscle contraction regulator (*tnt-4*) and 11 others, including pud-genes (S7 Table). Genes *cyp-33C8*, *cyp-34A9*, *dod-3*, *F40C5*.*2*, *hsp-16*.*2*, *hsp-16*.*41*, *pud-1*.*2*, *pud-2*.*1*, *pud-3*, and *pud-4* were the ten most significantly (adjusted *p*<0.001) expressed at the VL locus. Of the 10 genes, seven (*cyp-34A9*, *dod-3*, *F40C5*.*2*, *pud-1*.*2*, *pud-2*.*1*, *pud-3* and *pud-4)* were downregulated between 1.6 and 4.4-fold while the rest were upregulated on average 2.3-fold (Fig 6). Six DEGs mapped to the VC locus (Fig 6), which comprised of three stress response genes (*F31F7*.*1*, *mtl-1*, *clec-7*), a transport-related gene (*col-143*), a metabolic gene (*lipl-4*), and an unknown gene (S7 Table). Genes *F31F7*.*1*, *mtl-1*, *clec-7*, *lipl-4*, and *spp-27* were downregulated between 1.5 and 3.6-fold, with the exception of *col-143*, which was upregulated 2.4-fold (Fig 6 & S7 Table). Seven DEGs mapped to the VR locus (Fig 6) composed of a GluCl subunit (*glc-1*), a transporter (*F11A5*.*9*) and five unknown genes (S7 Table). The expression of the genes *glc-1*, *F11A5*.*9*, and *oac-18* was decreased by 1.4-fold, 1.4-fold, and 2.6-fold, respectively. The remaining genes had 1.54- to 10-fold higher expression levels (Fig 6).

A list of candidate genes for further study (S9 Table) has been created, including genes with an opposite expression between N2 and DA1316 *C*. *elegans* strain, genes within Abamectin-QTL, exclusive genes in DA1316 strain, and other putative drug-responsive genes.

## 4 Discussion

Parasitic nematodes pose a threat to human and animal health [2, 3], while the development of resistance against MLs such as IVM exacerbates the problem [16, 17, 63]. Therefore, it is crucial to understand the molecular mechanisms underlying ML resistance. In this study, we investigated gene expression in wild-type (N2) IVM-exposed *C*. *elegans* adults at maximum reproduction to understand the transcriptomic response to IVM exposure. Furthermore, the transcriptomic data was compared to microarray data from the IVM-resistant *C*. *elegans* (DA1316) strain [34], and the recently identified Abamectin-QTL [35]. We identified 615 differentially expressed genes in the wild-type *C*. *elegans* (N2) strain following exposure to IVM, of which 95% occurred in the IVM 10^−7^ M concentration. Only 31 N2 strain DEGs overlapped with the IVM-resistant *C*. *elegans* (DA1316) strain, 19 of which, including folate transporter FOLT-2 (*folt-2*) and transmembrane transporter T22F3.11 *(T22F3*.*11)*, displayed an opposite expression in either strain. Eighteen of the overlapping genes were enriched for metabolic Phase II enzymes, transmembrane transport, and stress response processes. In addition, we identified 45 differentially expressed genes that mapped within the Abamectin-QTL on *C*. *elegans* chromosome V, nine of which overlapped with DEGs from the IVM-resistant *C*. *elegans* (DA1316) strain. Following comprehensive analysis we identified potential candidate genes, T-type calcium channel CCA-1 (*cca-1*), potassium chloride cotransporter KCC2 (*kcc-2*), folate transporter FOLT-2 (*folt-2*) and alpha subunit of glutamate-gated channel GluCl-1 (*glc-1*) for further investigation with possible involvement in IVM resistance.

The ML drug target *glc-1* and the putative drug target *lgc-26* encoding ion channel subunits were downregulated in the IVM 10^−7^ M concentration. The significance of *glc-1* in ML response is evident through RNAi knockdown and loss-of-function-mutations [26, 36, 64, 65]. Heterologous expression of *C*. *elegans glc-1* and formation of a functional chloride channel has been reported in Xenopus oocytes [66]. The role of *lgc-26* in ML response remains unknown in *C*. *elegans*, but other cys-loop GABA receptor members have been implicated in the ML response of parasitic nematodes. For example, *lgc-37* was upregulated in the horse roundworm parasite, *Parascaris univalens* after IVM exposure [41] and the *lgc-54* in the sheep parasite *Teladorsagia circumcincta* was reported as potential candidate in IVM resistance through genome-wide studies [67]. However, Evans, Wit [35] recently challenged this claim, as they observed that *lgc-54* mutants did not display a competitive advantage over wild-type in control conditions.

We observed a downregulation of the calcium channel alpha subunit (*cca-1*) and potassium chloride cotransporter (*kcc-2*) genes after IVM exposure. Gene *cca-1* regulates pharyngeal pumping by facilitating the effective start of action potentials by influx of calcium ions in response to marginal cell motor neuron stimulation in *C*. *elegans* [68, 69]. A loss of function in *cca-1* has been reported to significantly reduce pharyngeal pumping [69]. Furthermore it is established that IVM reduces pharyngeal pumping in *C*. *elegans* by allosterically modulating GluCl hence increased influx of chloride ions [9]. Hypothetically, a pharyngeal pump regulator such as *cca-1* would be upregulated in response to decreased pharyngeal pumping rate, however, our results showed the opposite trend. Alternatively, the downregulation of *cca-1* could be a downstream effect, a consequence of the negative membrane potential induced by the chloride ion influx, or a defense/ protective mechanism aimed at reducing the adverse effects of IVM. Nonetheless, this highlights the importance of *cca-1* in IVM response in *C*. *elegans*. The *kcc-2* gene encodes a potassium chloride cotransporter, which, in conjunction with the sodium-driven chloride-bicarbonate transporter *abts-1*, mediates inhibitory GABA signaling. Mediation involves control of cellular chloride gradient to maintain membrane potential in neurons that control locomotion [70]. In addition, *kcc-2* and *abts-1* mutant of *C*. *elegans* were reported to exhibit paralysis after exposure to a GABA receptor agonist, muscimol [70]. Considering that IVM agonistically mediates GABA signaling through the inflow of chloride ions to cause paralysis [9], the role of *kcc-2* in IVM response is unknown and warrants exploration. Overall, these findings shed light on additional channels, such as *cca-1* and *kcc-2*, which could be investigated further as potential candidates for involvement in IVM resistance.

Seven of the 11 differentially expressed Transcription factors were downregulated and predominantly enriched for NHRs, which included *nhr-17*, *nhr-21*, *nhr-55*, *nhr-57*, *nhr-99*, *nhr-115*, *nhr-119*, *nhr-121*, *nhr-137* and *nhr-173*. Although NHRs have generally been suggested to regulate expression of detoxification genes (phase I, II and III), only a handful have experimental evidence (see review [61]). To our knowledge, none of the NHRs in the current study has been implicated in xenobiotic detoxification regulation. However, a notable example, *nhr-8* upregulation has been reported to increase expression of detoxification genes *cyp14A2*, *cyp14A5* and *cyp37B1*, and Pgp genes (*pgp-1*,*-3*,*-6*,*-*9 and -*13*) resulting in the reduced IVM efficacy [71]. Recent work by Guerrero et al. [72] emphasized this phenomenon, demonstrating *nhr-8* role in upregulation of Pgp genes (*pgp-3*, *-5*, *-11* and *-13*) in the presence of the drug tunicamycin. The GATA-type transcription factor, *elt-2* was downregulated in the current study. The proposed genes regulated by *elt-2* in *C*. *elegans* include those involved in protection against xenobiotic compounds: CYPs, GSTs, and UGTs such as *ugt-22* [73]. While it is conceivable that this downregulation of *elt-2* might explain the previously described downregulation of *ugt-22*, it is unclear why *elt-2* is downregulated in the presence of a xenobiotic such as IVM.

In this study, an analysis of putative apoptotic genes showed that four out of the five genes identified were downregulated, including C2H2-type zinc finger transcription factor *ces-1* and basic region leucine-zipper (bZIP) transcription factor *ces-2*. These genes have been previously shown to act as repressors of the key apoptotic activator *egl-1* under anti-apoptotic conditions. Under pro-apoptotic conditions, *egl-1* represses *ced-9*, thereby activating the pro-apoptotic genes *ced-3* and *ced-4*, hence apoptosis. The downregulation of *ces-1* and *ces-2* in this study is indicative of *egl-1* being liberated and subsequently able to repress *ced-9*, thereby activating the pro-apoptotic genes *ced-3* and *ced-4*, leading to apoptosis [62]. Additionally, caudal-type homeodomain transcription factor *pal-1*, a reported activator of *ced-3* [74], was found to be upregulated. To further support the pro-apoptotic response hypothesis, genes *ces-1*, *ces-2*, and *pal-1* were predominantly present in the highest IVM concentration (10^−7^ M), in which all worms were previously reported to be immobile [41]. While these findings suggest that IVM at concentrations of 10^−7^ M and 10^−8^ M partially activates the apoptotic machinery in the N2 *C*. *elegans* strain, it is important to note that other mechanisms involving above genes may also be at play as not all relevant apoptotic genes were detected in the data.

Our second objective was to compare *C*. *elegans* strains N2 (wild-type) and DA1316 (IVM-resistant) following IVM exposure. Comparing the RNAseq and microarray data from the N2 and DA1316 strains, respectively, revealed that the N2 strain had four times the number of differentially expressed genes with only a 4% (31 genes) overlap between the two strains. Nineteen of these overlapped genes displayed an opposite expression and may have a role in IVM metabolism or resistance. For example, the folate transporter *folt-2*, whose expression was reduced 6-fold and 2-fold in the resistant DA1316 strain, but increased 4-fold in the sensitive N2 strain, may be involved in the import of IVM. Another potential candidate is the transmembrane transporter *T22F3*.*11*, whose expression increased by 3-fold in the resistant strain, but decreased 7-fold in the sensitive N2 strain, suggesting a role in IVM efflux. In addition, genes are that are exclusively expressed in the resistant strain could also be an important group for further exploration. Overall, further studies are needed to confirm the role of these genes in IVM response. Twelve of the 31 overlapping genes were downregulated, including four pud-genes, *pud-2*.*1*, *pud-3*, and *pud-4*. Pud-genes are unique to *Caenorhabditis* spp. without known orthologues in other nematodes and their function remains elusive. In spite of that, Cui et al. [75] reported slow growth and hypersensitivity to cadmium of *C*. *elegans* after *pud-4* (*F15E11*.*12*) RNAi knockdown. Another study reported downregulation of *pud-1*.*2* and *pud-4* following viral infection of *C*. *elegans* [76]. Other overlapping genes such as *hsp-17* encoding a heat shock protein, exhibited inverted regulation in either strain. Gene *hsp-17*, a stress responder, was downregulated 1.7-fold in DA1316 and upregulated 4-fold in N2 strain. This re-emphasizes the inherent sensitivity of the N2 strain to IVM than its resistant or tolerant counterpart. The discrepancy in gene expression between the two studies could be attributable to strain, technique, life-stage, or IVM concentration. Strain differences may account for most of the difference in DEGs, since N2 is more sensitive to IVM than resistant or tolerant strains [34, 77]. Thus, the N2 strain may have more elevated cellular and biological processes, which could account for the increase in DEGs. Overall, we speculate that pud-genes may be universal stress responders exclusively in *C*. *elegans*.

As a final objective, we mapped the 615 DEGs derived from the IVM-exposed *C*. *elegans* to the recently identified Abamectin-QTL [35]. We identified 45 DEGs that corresponded to the QTL regions and compared them to the proposed list of candidate genes from the QTL [35], but only two genes, putative folate transporter (*folt-2*) and *dod-3*, between the two data sets overlapped. Gene *folt-2* supposedly participates in the active uptake of folate [78], a B-class vitamin central in the synthesis of nucleotides and amino acids [79]. Like other eukaryotes, *C*. *elegans* are intrinsically deficient in folate [80], and therefore rely on dietary sources such as OP50 *E*. *coli*. In our data, *folt-2* was upregulated, which may be caused by restriction in dietary intake due IVM-induced decreased pharyngeal pumping [9]. This could lead to folate deficiency in the worm. Whether this folate deficiency triggers upregulation of *folt-2* remains unclear and therefore entails further study. In contrast, the function *dod-3* and its role ML response is unknown. Overall, the role of *folt-2*, *dod-3* and other 43 genes that corresponded to the Abamectin-QTL in ML response is unknown and prompts further investigation.

In conclusion, we have profiled the transcriptomes of adult wild-type (N2) *C*. *elegans* after exposure to different IVM concentrations and revealed predominantly downregulated diverse sets of genes. Some genes overlapped with a previous microarray study on IVM-resistant *C*. *elegans* while others mapped to recently described abamectin-QTL. Based on this, we have a compiled a list of potential candidate genes for further investigation. Although Abamectin and Ivermectin (IVM) belong to the same subgroup of avermectins, it is important to exercise caution when making comparisons between the two drugs. While Abamectin serves as a precursor drug for IVM, the pharmacokinetics of these drugs have been reported to be considerably different [81, 82]. These differences have been attributed, in part, to variations in the drugs’ lipophilicity [83]. Future studies employing quantitative genetics approaches, such as identifying IVM-QTL and corresponding candidate genes, validated through the use of near-isogenic lines, mutant strains, and transcriptomic profiling, would provide more detailed information.

## Supporting information

S1 Fig Differential gene expression in IVM-exposed *C*. *elegans* DA1316 strain. Venn diagrams showing the number of DEGs in *C*. *elegans* DA1316 strain between IVM concentrations 10^−6^ M and 10^−7^ M.
(TIF)

S1 Table Differential gene expression in *C*. *elegans* (N2) at 10^−7^ M or 10^−8^ M IVM. (XLSX)

S2 Table Differential gene expression in *C*. *elegans* (N2) at 10^−7^ M compared to 10^−8^ M IVM. (XLSX)

S3 Table Top 10 up—and downregulated differentially expressed genes in *C*. *elegans* (N2) at 10^−7^ M and 10^−8^ M IVM. (DOCX)

S4 Table Over-representation analysis of IVM-exposed *C*. *elegans* (N2) differentially expressed genes. (XLSX)

S5 Table Differential gene expression in *C*. *elegans* DA1316 vs N2 after IVM exposure. (XLSX)

S6 Table Enrichment analysis of the differentially expressed genes from IVM-exposed *C*. *elegans* (N2) across all chromosomes. (XLSX)

S7 Table Differentially expressed genes from IVM-exposed *C*. *elegans* (N2) strain mapped to Abamectin-QTL. (XLSX)

S8 Table Differentially expressed genes from IVM-exposed *C*. *elegans* (DA1316) strain mapped to Abamectin-QTL. (XLSX)

S9 Table A list of potential candidate genes for further investigation. (XLSX)
