# Supplementary figures and images for: Transcriptomics of ivermectin response in Caenorhabditis elegans: Integrating abamectin quantitative trait loci and comparison to the Ivermectin-exposed DA1316 strain
Source: PLoS One. 2023 May 4;18(5):e0285262. doi: 10.1371/journal.pone.0285262 (PMC10159168; doi:10.1371/journal.pone.0285262)

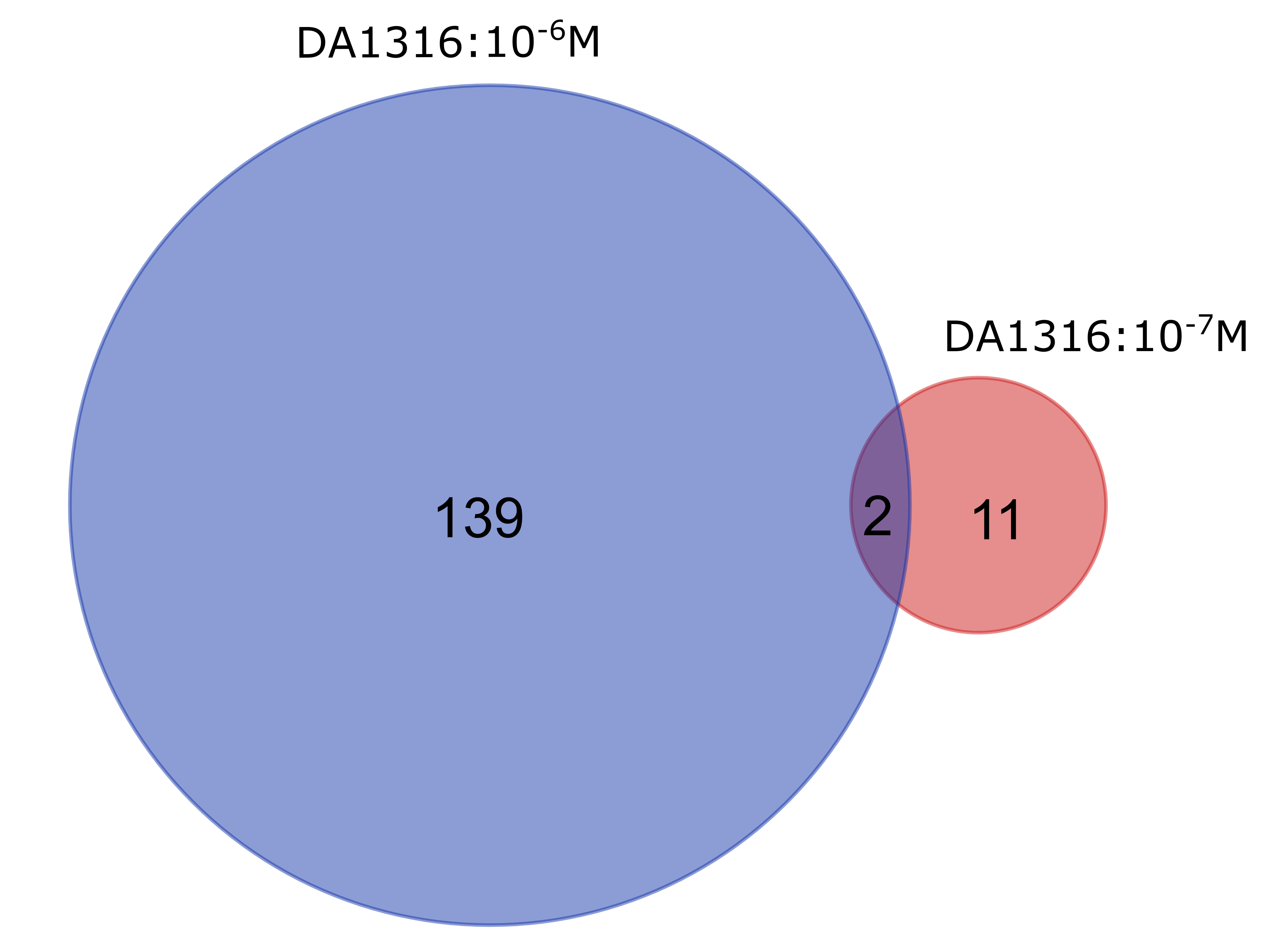

Supplement: S1 Fig — Venn diagrams showing the number of DEGs in C. elegans DA1316 strain between IVM concentrations 10−6 M and 10−7 M. (TIF) [file pone.0285262.s001.tif]
