# Supplementary material for: Transcriptomics of ivermectin response in Caenorhabditis elegans: Integrating abamectin quantitative trait loci and comparison to the Ivermectin-exposed DA1316 strain
Source: PLoS One. 2023 May 4;18(5):e0285262. doi: 10.1371/journal.pone.0285262 (PMC10159168; doi:10.1371/journal.pone.0285262)
Supplement: S3 Table — (DOCX) [file pone.0285262.s004.docx]

Table S1. Top 10 up - and downregulated differentially expressed genes in C. elegans (N2) after exposure to 10^-7^ M and 10^-8^ M IVM

| IVM concentration | Gene | Log2FoldChange | | -Log (Adj. p-value) | Description* |
| --- | --- | --- | --- | --- | --- |
| 10^-7^ M | *M03A8.3* | 6.27 | | 1.8 | PH-like domain superfamily |
|  | *Y49G5A.1* | 3.71 | | 18.5 | serine-type endopeptidase inhibitor activity |
|  | *F57G8.7* | 3.36 | | 12.3 | Enriched in structures including cephalic sheath cell, coelomocyte, neurons, and ventral nerve cord |
|  | *F25E5.5* | 3.35 | | 1.4 | protein containing a F-box motif |
|  | *C49G7.12* | 3.19 | | 1.7 | Enriched in body wall muscle, cephalic sheath, neurons, coelomycte and intestine |
|  | *K03D3.2* | 3.15 | | 10.8 | Enriched in structures including cephalic sheath cell, coelomocyte, neurons, and ventral nerve cord |
|  | *B0205.13* | 2.98 | | 3.4 | small, novel protein conserved in C. remanei and C. briggsae |
|  | *irg-1* | 2.90 | | 3.5 | DUF1768-domain protein |
|  | *irg-2* | 2.69 | | 2.6 | DUF1768-domain protein |
|  | *ZK228.4* | 2.58 | | 2.9 | acetyltransferase activity and immune response |
|  | *K07F5.22* | -10.81 | | 2.5 | ortholog of human TMEM167B (transmembrane protein 167B) |
|  | *nsy-4* | -5.37 | | 2.2 | PMP22/EMP/claudin/calcium channel gamma subunit |
|  | *C23G10.11* | -3.60 | | 3.8 | Expressed in hypodermal cell |
|  | *C10B5.3* | -3.04 | | 2.0 | affected by twelve chemicals including rotenone; D-glucose; and Zidovudine based on RNA-seq and microarray studies |
|  | *T07G12.5* | -2.89 | | 10.8 | L-ascorbic acid transmembrane transporter activity |
|  | *T22F3.11* | -2.81 | | 13.6 | transmembrane transporter activity |
|  | *dhs-7* | -2.60 | | 6.5 | involved in regulation of reactive oxygen species metabolic process |
|  | *pud-3* | -2.13 | | 8.7 | protein with the following domains: Up-Regulated in long-lived daf-2 and Up-regulated in Daf-2 |
|  | *ftn-1* | -1.98 | | 16.0 | ferritin heavy chain homologs |
|  | *poml-4* | -1.88 | | 3.3 | enable arylesterase activity |
| 10^-8^ M | *atp-6* | 2.06 | | 42.2 | ATP synthase subunit a |
|  | *hsp-70* | 1.57 | | 1.7 | heat shock protein |
|  | *hsp-16.11* | 1.25 | | 1.4 | heat shock protein |
|  | *hsp-16.2* | 0.95 | | 1.7 | heat shock protein |
|  | *F44E5.4* | 0.95 | | 1.7 | heat shock protein |
|  | *hsp-16.41* | 0.87 | | 1.4 | heat shock protein |
|  | *ath-1* | 0.84 | | 2.4 | enable carboxylic ester hydrolase activity and palmitoyl-(protein) hydrolase activity |
|  | *pqn-31* | 0.65 | | 2.1 | affected by twenty chemicals including Ethanol; methylmercuric chloride; and rotenone based on RNA-seq and microarray studies |
|  | *coa-1* | 0.65 | 8.1 | | involved in mitochondrial cytochrome c oxidase assembly and mitochondrial respiratory chain complex I assembly |
|  | *Y94H6A.10* | 0.64 | 3.8 | | affected by twenty-four chemicals including 1-methylnicotinamide; Tunicamycin; and D-glucopyranose based on RNA-seq and microarray studies |
|  | *mvk-1* | -1.02 | 6.5 | | Mevalonate kinase (mvk) |
|  | *let-413* | -0.92 | 3.7 | | human-like ERBIN |
|  | *npa-1* | -0.84 | 1.3 | | precursor protein that is cleaved to strong binding peptides for fatty acids and retinol, and also acts as a carrier protein for these lipids within nematodes and secreted by parasitic nematode species |
|  | *cyp-35A2* | -0.84 | 6.3 | | cytochrome P450s |
|  | *myrf-2* | -0.78 | 1.4 | | human-like myelin regulatory factor |
|  | *wht-7* | -0.74 | 1.4 | | ABC-type transporter |
|  | *mlt-7* | -0.73 | 2.2 | | heme peroxidase |
|  | *dod-3* | -0.70 | 6.0 | | integral component of membrane |
|  | *kin-4* | -0.66 | 4.2 | | protein serine/threonine kinase activity |
|  | *ugt-22* | -0.61 | 1.4 | | UDP-glycosyltransferase |

*Full description can be found in Table S1
